# House dust mite-induced autophagy dysregulation and apoptosis in bronchial epithelial injury in asthma

**DOI:** 10.3389/fped.2026.1922943

**Published:** 2026-08-24

**Authors:** Jun Wang, Baolei Wang, Lu Chen

**Affiliations:** Department of Pediatrics, Jiading Branch of Shanghai General Hospital, Shanghai Jiao Tong University School of Medicine, Shanghai, China

**Keywords:** airway epithelial injury, apoptosis, asthma, autophagy, house dust mite

## Abstract

**Introduction:**

Airway epithelial injury is a central feature of asthma and contributes to persistent airway inflammation, mucus overproduction, and airway remodeling. House dust mite (HDM) is a major aeroallergen associated with allergic asthma, yet the intracellular stress responses underlying HDM-induced epithelial damage remain incompletely understood. Here, we investigated changes in the autophagic process and apoptotic signaling during HDM-induced airway injury using both *in vivo* and *in vitro* models.

**Methods:**

Wild-type BALB/c mice were exposed to HDM by intranasal administration, and airway pathology was evaluated using hematoxylin and eosin staining (H&E) and periodic acid-Schiff (PAS) staining. BEAS-2B bronchial epithelial cells were treated with HDM *in vitro*, followed by transmission electron microscopy (TEM), immunofluorescence (IF) staining, and Western blot to assess ultrastructural changes and the expression of autophagy- and apoptosis-related proteins.

**Results:**

HDM exposure induced asthma-like airway pathology in mice, characterized by peribronchial inflammatory infiltration and increased mucus production. In bronchial epithelial cells, HDM caused mitochondrial ultrastructural injury and autophagosome accumulation, suggesting disruption of epithelial intracellular homeostasis. Further analyses showed increases in LC3-related signals and p62 levels, suggesting dysregulated autophagic processing. These autophagy-related alterations were accompanied by activation of apoptotic signaling.

**Discussion:**

Taken together, our findings indicate that HDM-induced airway epithelial injury is accompanied by autophagy dysregulation and activation of apoptotic signaling. These results provide insight into epithelial stress responses involved in allergic airway inflammation and highlight autophagic dysregulation and apoptosis as cellular events associated with HDM-related bronchial epithelial damage.

## 1 Introduction

Asthma remains one of the most common chronic inflammatory airway diseases and is characterized by airway hyperresponsiveness, recurrent respiratory symptoms, and variable airway obstruction (1). Although current therapies have improved symptom control in many patients, asthma is still driven by complex and incompletely resolved interactions between environmental exposures, airway inflammation, and structural changes in the airway wall. Among the cellular compartments involved in this process, the airway epithelium occupies a particularly important position because it forms the first interface between inhaled environmental stimuli and the host airway response. The airway epithelium is no longer viewed simply as a passive physical barrier. Instead, it is increasingly recognized as an active regulator of airway homeostasis and inflammation (2). Epithelial cells maintain barrier integrity, sense inhaled allergens and irritants, and release mediators that shape downstream immune responses. When epithelial homeostasis is disrupted, barrier dysfunction, persistent inflammation, mucus overproduction, and airway remodeling may follow. Epithelial injury and epithelial cell death have therefore been considered important pathological components of asthma, particularly in relation to chronic airway inflammation and structural remodeling (3).

House dust mite (HDM) is one of the most clinically relevant inhaled allergens associated with allergic asthma. HDM exposure can directly affect airway epithelial cells, impair epithelial barrier function, and amplify inflammatory responses, thereby contributing to asthma exacerbation and disease persistence (4, 5). However, the intracellular events that translate HDM exposure into epithelial cell injury remain insufficiently defined. Clarifying these events is important because epithelial damage is not merely a consequence of inflammation but may also actively reinforce airway inflammation and remodeling.

Autophagy is a conserved intracellular process responsible for the degradation of damaged organelles and misfolded or aggregated proteins, thereby preserving cellular homeostasis (6). Its protective role depends on the integrity of the entire autophagic flux, including autophagosome formation, fusion with lysosomes, and degradation of sequestered cargo. When this process is interrupted, autophagy substrates such as p62 and damaged organelles may accumulate within cells, converting an adaptive stress response into a source of cellular injury (6, 7). In several respiratory diseases, including bronchopulmonary dysplasia and chronic obstructive pulmonary disease, impaired autophagic clearance has been linked to epithelial injury, apoptosis, and disease progression (7–9). Despite these observations, whether autophagic dysregulation is associated with HDM-induced epithelial injury in asthma remains unclear. In particular, it is not well established whether HDM exposure primarily activates autophagosome formation as part of an adaptive epithelial response, or whether it disrupts downstream autophagic degradation and thereby promotes cellular damage. The relationship between HDM-induced autophagic disturbance and bronchial epithelial apoptosis therefore represents an important unresolved issue in asthma pathobiology.

Based on this background, we hypothesized that HDM-induced bronchial epithelial injury would be associated with disrupted autophagic homeostasis and activation of epithelial apoptosis. To test this hypothesis, we used an HDM-induced mouse model of allergic airway inflammation together with HDM-exposed BEAS-2B bronchial epithelial cells. Airway pathological changes, epithelial ultrastructural alterations, and the expression of key autophagy- and apoptosis-related proteins were examined. This study aimed to characterize autophagy-related alterations and apoptotic signaling during HDM-induced bronchial epithelial injury, thereby providing insight into the epithelial stress responses associated with asthma.

## 2 Materials and methods

### 2.1 Animals

Specific pathogen-free female BALB/c mice aged 6–8 weeks and weighing 20–22 g were purchased from Shanghai PHENOTEK Biotechnology Co., Ltd. The mice were housed under specific pathogen-free conditions. All animal procedures were approved by the Institutional Animal Care and Use Committee (approval no. AUP-202604-01).

BALB/c mice were randomly assigned to the control or HDM group. HDM extract was obtained from Stallergenes Greer (XPB91D3A2.5). An HDM-induced asthma model was established by intranasal administration as previously described (10, 11). During the sensitization phase, mice in the HDM group received intranasal instillation of 20 μL HDM solution at a concentration of 1 μg/μL on days 0 and 1, whereas mice in the control group received an equal volume of sterile PBS. During the challenge phase, mice in the HDM group were challenged intranasally with 20 μL HDM solution (1 μg/μL) once daily for three consecutive days on days 11, 12, and 13. Mice in the control group received an equal volume of sterile PBS according to the same schedule. Twenty-four hours after the final challenge, mice were euthanized by gradual-fill CO_2_ inhalation, followed by cervical dislocation to confirm death. The left lung tissues were collected and fixed in 4% paraformaldehyde for subsequent histological analysis, whereas the right lung tissues were snap-frozen in liquid nitrogen and stored at −80 °C until further use.

### 2.2 Cell culture

The human bronchial epithelial cell line BEAS-2B was purchased from IMMOCELL Biotechnology Co., Ltd and cultured in the recommended BEAS-2B medium (IM-H128-1; IMMOCELL). Cells were maintained at 37 °C in a humidified atmosphere with 5% CO_2_. Experiments were performed using cells in the logarithmic growth phase. Cells were allocated to control or HDM-exposed groups. For HDM exposure, cells were treated with HDM extract (100 μg/mL) for 24 h, with reference to previously reported HDM-induced airway epithelial cell models (12, 13). Control cells were maintained in complete medium without HDM for the same duration.

### 2.3 Histological analysis of lung tissues

Left lung tissues fixed in 4% paraformaldehyde were dehydrated, cleared, embedded in paraffin, and sectioned at a thickness of 4 μm. Hematoxylin and eosin (H&E) staining and periodic acid–Schiff (PAS) staining were performed according to standard protocols.

For H&E staining analysis, peribronchial inflammatory cell infiltration was evaluated under a light microscope and scored as previously described (14). Briefly, inflammation was graded on a 0–4 scale: 0, no inflammatory cell infiltration around the airway; 1, a small number of inflammatory cells around the airway; 2, one layer of inflammatory cells surrounding the airway; 3, two to four layers of inflammatory cells surrounding the airway; and 4, more than four layers of inflammatory cells surrounding the airway.

For PAS staining analysis, mucus production in the airway epithelium was assessed microscopically (15, 16). The total tissue area and PAS-positive area in each field were quantified using Image-Pro Plus 6.0 software. The percentage of PAS-positive mucus area was calculated as follows: PAS-positive area (%) = PAS-positive area/total tissue area in the field × 100%.

### 2.4 Transmission electron microscopy (TEM)

After 24 h of HDM exposure, BEAS-2B cells were fixed in 2.5% glutaraldehyde at 4 °C overnight and post-fixed with 1% osmium tetroxide. The samples were dehydrated in a graded acetone series, embedded in epoxy resin, and cut into 60–80-nm ultrathin sections. After staining with uranyl acetate and lead citrate, the sections were examined under a transmission electron microscope to assess cellular ultrastructure, with particular attention to mitochondrial morphology and autophagosome abundance. For quantitative analysis, autophagosomes were identified according to their characteristic double-membrane ultrastructure and counted in randomly selected cells examined at the same magnification. Three independent experiments were performed.

### 2.5 Immunofluorescence (IF) staining

BEAS-2B cells were seeded on coverslips in 12-well plates. After HDM exposure, cells were fixed with 4% paraformaldehyde at room temperature for 15 min, permeabilized with 0.1% Triton X-100 for 15 min and blocked with 5% bovine serum albumin for 1 h. The cells were then incubated overnight at 4 °C with primary antibodies against LC3 (1:100; 14600-1-AP, Proteintech) or cleaved caspase-3 (1:100; 9661T, Cell Signaling Technology). After washing with PBS, cells were incubated with the appropriate fluorophore-conjugated secondary antibodies (1:500; HA1121 or HA1122, HUABIO) for 1 h at room temperature in the dark. Nuclei were counterstained with DAPI (P0131, Beyotime), and images were acquired using a confocal microscope. The fluorescence intensity was quantified using ImageJ software. Three randomly selected fields were analyzed for each biological sample, and the average value was used for statistical analysis.

### 2.6 Mitochondrial membrane potential assay using JC-1 staining

BEAS-2B cells were seeded in glass-bottom confocal dishes. After HDM exposure, mitochondrial membrane potential was assessed using a JC-1 assay kit (C2006, Beyotime) according to the manufacturer's instructions. Cells were incubated with JC-1 staining working solution at 37 °C for 20 min in the dark and then washed twice with JC-1 staining buffer. After replacement with complete culture medium, fluorescence images were acquired using a confocal microscope. Fluorescence images were analyzed using ImageJ software, and three randomly selected fields per biological sample were quantified. The average value was used for statistical analysis.

### 2.7 Western blot

Cells were lysed in pre-chilled RIPA lysis buffer (PC101, Epizyme) containing protease and phosphatase inhibitors (P002, NCM). Cells were lysed on ice for 30 min, followed by centrifugation at 12,000 rpm for 15 min at 4 °C. The supernatants were carefully collected to obtain total cellular protein. Protein concentrations were measured using a BCA protein assay kit (ZJ101, Epizyme). Protein samples were mixed with loading buffer (NP0007, Invitrogen) and denatured at 100 °C for 10 min. Equal amounts of protein (20 μg per lane) were separated by SDS-PAGE and transferred onto PVDF membranes. After transfer, the membranes were blocked with 5% non-fat milk in TBST for 1.5 h at room temperature and then incubated with the corresponding primary antibodies overnight at 4 °C. After washing with TBST, the membranes were incubated with secondary antibody for 1 h at room temperature. Protein signals were detected using an enhanced chemiluminescence kit and captured with a chemiluminescence imaging system. Exposure times were optimized in preliminary experiments to avoid signal saturation and to ensure that the detected signals remained within the linear dynamic range.

The following antibodies were used: LC3 (1:2,000; 14600-1-AP, Proteintech), p62 (1:5,000; 84826-1-RR, Proteintech), Bax (1:20,000; 50599-2-Ig, Proteintech), Bcl-2 (1:2,000; HA723111, HUABIO), cleaved caspase-3 (1:1,000; 9661T, Cell Signaling Technology), β-actin (1:40,000; EM21002; HUABIO), and HRP-conjugated secondary antibodies (1:50000; HA1001; 1:20,000, HA1006, HUABIO).

### 2.8 Statistical analysis

All statistical analyses were performed using GraphPad Prism 10. Data are presented as the mean ± standard deviation (SD). All quantitative outcomes, including histological analysis of lung tissues, were compared between the control and HDM groups using an unpaired two-tailed Student's *t*-test. A value of *p* < 0.05 was considered statistically significant.

## 3 Results

### 3.1 HDM exposure induced asthma-like airway inflammation and mucus overproduction in mice

**Figure 1:**
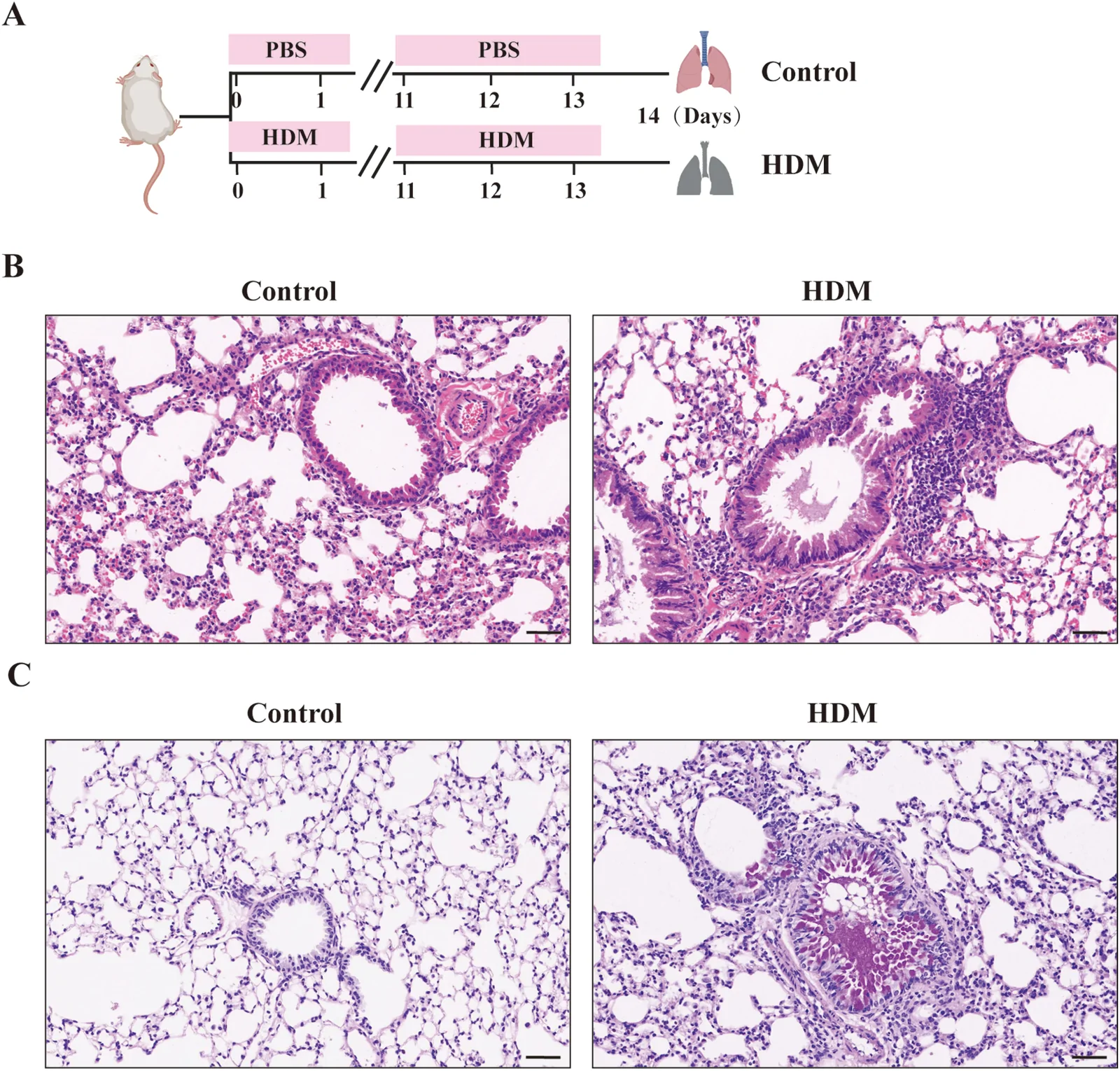
HDM exposure induced asthma-like airway inflammation and mucus overproduction in mice. **(A)** Schematic diagram of the HDM-induced asthma model. **(B)** Representative H&E-stained lung sections showing increased peribronchial inflammatory infiltration in HDM-exposed mice compared with controls (*n* = 4 per group). Scale bar, 50 μm. **(C)** Representative PAS-stained lung sections showing enhanced goblet cell hyperplasia and mucus production after HDM exposure (*n* = 4 per group). Scale bar, 50 μm.

Intranasal HDM exposure was used to induce an asthma-like airway phenotype in mice (Figure 1A). In H&E-stained lung sections, control mice showed preserved bronchial epithelial structure with little inflammatory cell infiltration around the airways or adjacent vessels. In contrast, HDM-exposed mice displayed epithelial hyperplasia, increased intraluminal secretions, and dense peribronchial inflammatory infiltration. Quantitative scoring confirmed that airway inflammation was significantly increased in the HDM group compared with the control group (1.50 ± 1.00 vs. 0, *p* < 0.05; Figure 1B). PAS staining further showed minimal goblet cell staining in control airways, whereas HDM exposure led to prominent goblet cell hyperplasia and abundant PAS-positive mucus within the airway lumen. The PAS-positive mucus area was significantly higher in the HDM group than in the control group (4.07 ± 1.25 vs. 0.05 ± 0.10, *p* < 0.001; Figure 1C). These findings indicate that HDM exposure induced an asthma-like airway phenotype in mice. Based on this *in vivo* phenotype, we next used an *in vitro* bronchial epithelial cell model to investigate the cellular changes associated with HDM-induced epithelial injury.

### 3.2 HDM exposure induced mitochondrial injury and autophagosome accumulation

**Figure 2:**
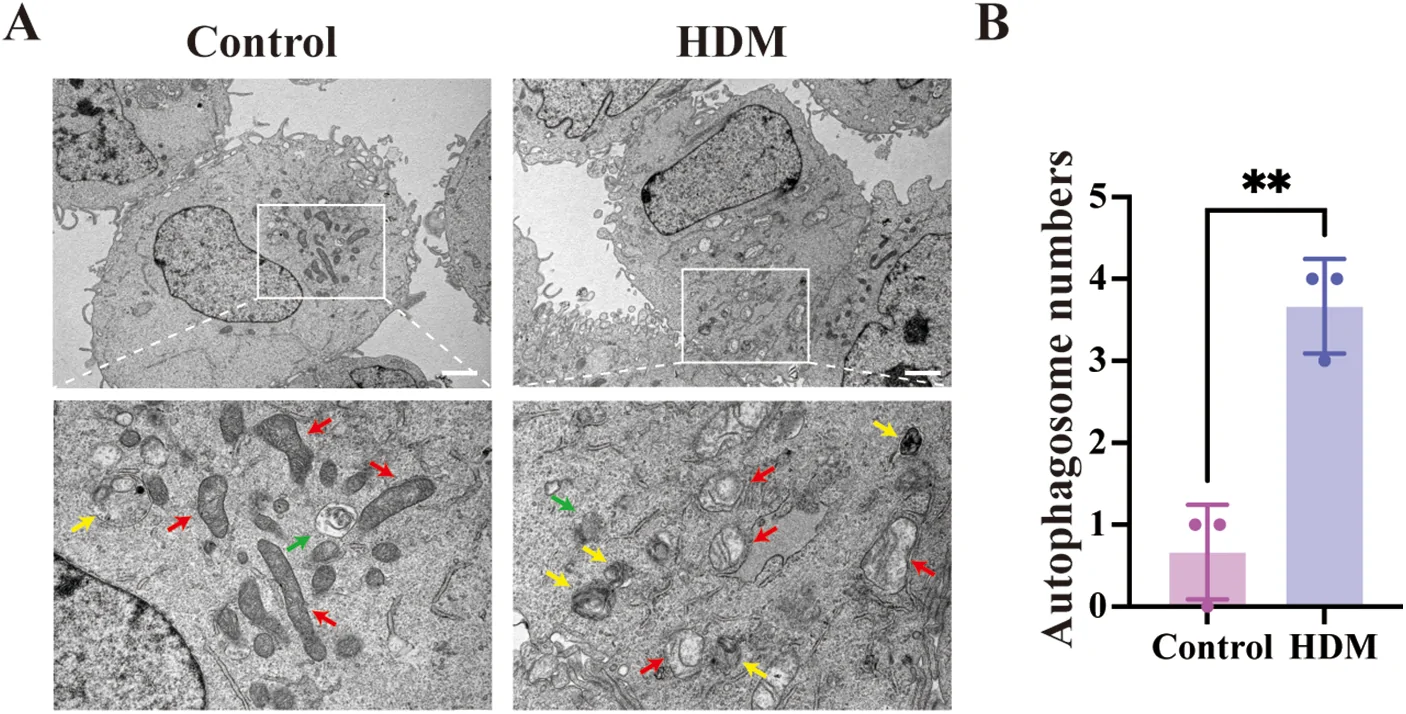
Mitochondrial abnormalities and autophagy-related structures in HDM-exposed bronchial epithelial cells. **(A)** Representative TEM images of BEAS-2B cells treated with or without HDM. Red arrows: mitochondria, green arrows: autolysosomes, yellow arrows: autophagosomes. Scale bar, 2 μm. **(B)** Quantification of the number of autophagosomes per cell (*n* = 3). Data are presented as mean ± SD. *p < 0.05, **p < 0.01, and ***p < 0.001; ns, not significant.

To further examine the cellular changes associated with HDM-induced epithelial injury, the ultrastructure of BEAS-2B cells was assessed by TEM. Control cells showed relatively preserved mitochondrial morphology and few intracellular autophagic structures. In contrast, HDM-exposed cells displayed marked mitochondrial swelling, with partial disruption or loss of cristae. Scattered autophagosomes and a small number of autolysosomes were also observed in the cytoplasm of HDM-exposed cells (Figures 2A,B). These ultrastructural findings suggest an association between HDM exposure, mitochondrial abnormalities, and changes in autophagy-related structures.

### 3.3 HDM exposure induced autophagy dysregulation in bronchial epithelial cells

**Figure 3:**
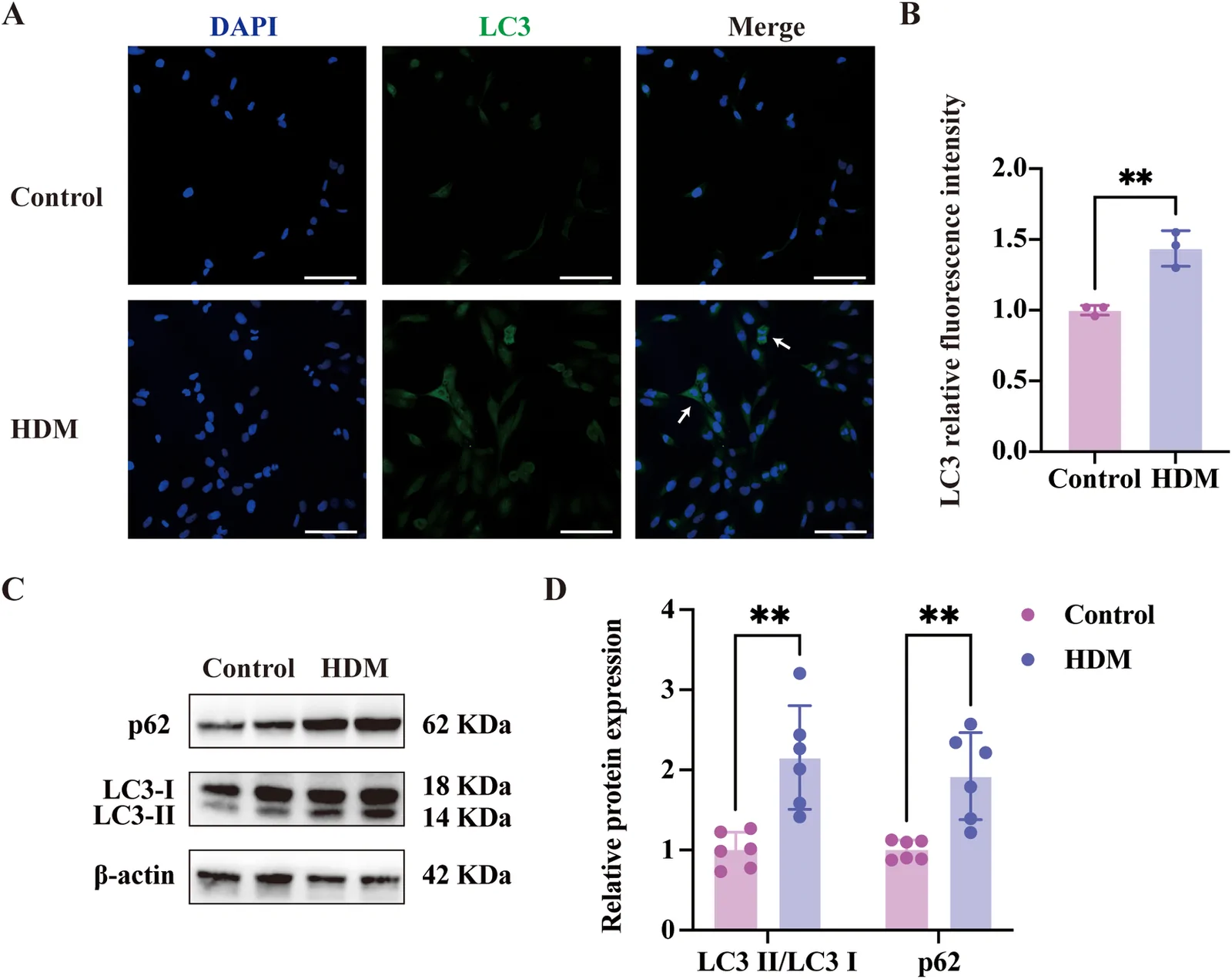
HDM exposure altered autophagy-related markers in bronchial epithelial cells. **(A,B)** Typical IF images and quantitative analysis of LC3 fluorescence intensity in BEAS-2B cells treated with or without HDM (*n* = 3). White arrows indicate LC3 puncta. Blue: DAPI, green: LC3. Scale bar, 100 μm. **(C,D)** Western blot analysis and quantification of autophagy-related proteins LC3-II/LC3-I and p62 in BEAS-2B cells. Densitometric analysis of Western blot bands, normalized to β-actin (*n* = 3). *p < 0.05, **p < 0.01, and ***p < 0.001; ns, not significant.

To further characterize the autophagy-related changes observed in HDM-exposed BEAS-2B cells, we examined the levels of autophagy-related proteins LC3 and p62. IF staining showed increased relative LC3 fluorescence intensity in HDM-exposed cells compared with controls (Figures 3A,B). Western blot further demonstrated a significant increase in the LC3-II/LC3-I ratio after HDM exposure. Meanwhile, the protein level of p62, a substrate degraded through the autophagy–lysosome pathway, was also significantly elevated (Figures 3C,D). These findings suggest dysregulated autophagic processing rather than simple activation of autophagy.

### 3.4 HDM exposure activated apoptotic signaling in bronchial epithelial cells

**Figure 4:**
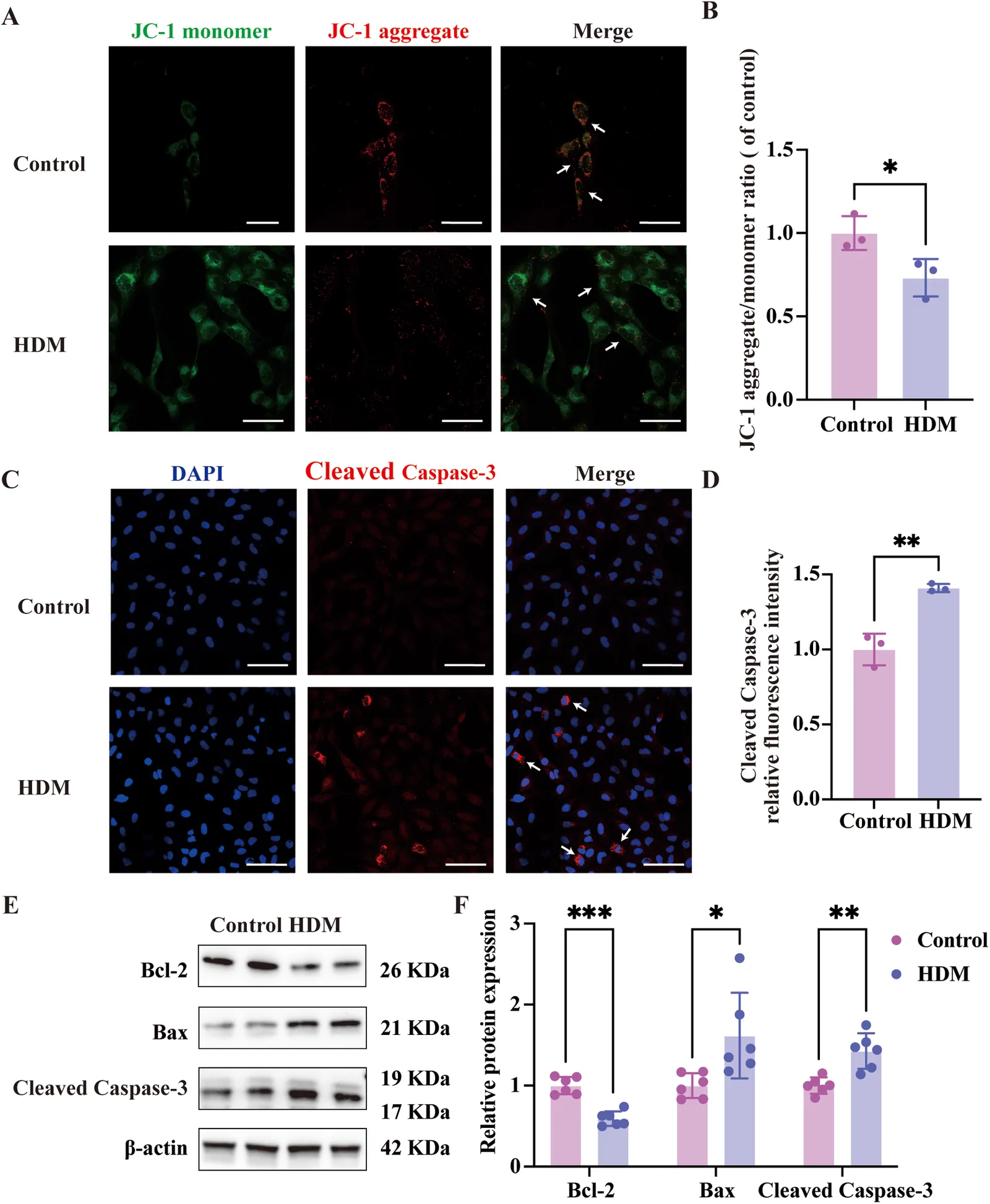
Activation of apoptotic signaling in HDM-exposed bronchial epithelial cells. **(A,B)** Representative images of JC-1 staining and quantitative analysis of the JC-1 aggregate/monomer fluorescence ratio in BEAS-2B cells treated with or without HDM (*n* = 3). Red: JC-1 aggregates, Green: JC-1 monomers. White arrows indicate representative cells. Scale bar, 50 μm. **(C,D)** Representative IF images and quantitative analysis of cleaved caspase-3 fluorescence intensity in BEAS-2B cells treated with or without HDM (*n* = 3). White arrows indicate cleaved caspase-3-positive signals. Blue: DAPI, red: cleaved caspase-3. Scale bar, 100 μm. **(E,F)** Western blot analysis and quantification of apoptosis-related proteins Bcl-2, Bax, and cleaved caspase-3 in BEAS-2B cells. Densitometric analysis of Western blot bands normalized to β-actin (*n* = 3). Data are presented as the mean ± SD. *p < 0.05, **p < 0.01, and ***p < 0.001; ns, not significant.

Impairment of autophagy can prevent the timely clearance of damaged organelles, including mitochondria, and often precedes the activation of apoptosis (9, 17). Moreover, our observations of TEM showed mitochondrial structural injury (Figure 2A). Given that mitochondrial membrane potential is closely associated with activation of the apoptotic pathway (18), JC-1 staining was performed to assess mitochondrial membrane potential in BEAS-2B cells. The results showed that HDM exposure significantly reduced the JC-1 aggregate/monomer ratio, indicating mitochondrial membrane depolarization (Figures 4A,B). We examined the expression of proteins involved in the apoptotic execution pathway. IF staining showed that cleaved caspase-3 signals were markedly enhanced in HDM-exposed BEAS-2B cells compared with control cells (Figures 4C,D). Western blotting further showed that, after 24 h of HDM exposure, the expression levels of Bax and cleaved caspase-3 were significantly increased, whereas the expression of the anti-apoptotic protein Bcl-2 was reduced (Figures 4E,F). Together, these results suggest a potential association between autophagy dysregulation and activation of apoptotic signaling in HDM-exposed bronchial epithelial cells.

## 4 Discussion

In this study, we investigated whether HDM-induced airway epithelial injury is associated with altered autophagic homeostasis and epithelial apoptosis. Using an HDM-induced mouse model of allergic airway inflammation together with HDM-exposed BEAS-2B bronchial epithelial cells, we found that HDM exposure induced asthma-like airway inflammation and mucus overproduction *in vivo* and was accompanied *in vitro* by mitochondrial structural damage, autophagosome accumulation, elevated LC3-II/LC3-I and p62 levels, and activation of apoptotic signaling. These findings suggest that autophagy dysregulation and epithelial apoptosis may be involved in HDM-induced airway epithelial injury.

The airway epithelium plays an active role in asthma pathobiology by regulating barrier integrity, immune responses, mucus production, and airway remodeling (19–21). Accordingly, epithelial injury is increasingly recognized as an important contributor to disease progression rather than merely a consequence of airway inflammation. In this context, our *in vivo* findings provide pathological evidence that HDM exposure induces two key features of allergic airway disease: inflammatory cell infiltration and mucus overproduction. More importantly, our subsequent *in vitro* experiments focused on bronchial epithelial cell responses, allowing us to examine the intracellular stress responses that may accompany HDM-related epithelial injury.

A major observation in this study was the accumulation of autophagosomes in HDM-exposed bronchial epithelial cells. Increased autophagosome abundance can either reflect enhanced autophagy initiation or impaired autophagic degradation. This distinction is important because autophagy is protective only when the process proceeds efficiently from autophagosome formation to lysosomal degradation (7, 22). Autophagy is defined as a dynamic degradation pathway that supports cellular renovation by removing damaged organelles and proteins (23). However, when autophagic clearance is incomplete, autophagy substrates such as p62 and damaged organelles may accumulate, turning an adaptive response into a source of cellular stress (9, 23). In the present study, HDM exposure increased LC3 fluorescence intensity and LC3-II/LC3-I levels, while p62 protein levels were also elevated. Because p62 is normally degraded through the autophagy–lysosome pathway, this pattern suggests dysregulated autophagic processing rather than simple activation of autophagy. This interpretation is supported by previous studies in respiratory epithelial injury. In COPD-related models, insufficient autophagy has been linked to epithelial senescence, protein aggregate accumulation, and disease progression (24). Similarly, Araya et al. found that insufficient autophagy was involved in idiopathic pulmonary fibrosis, a disease in which epithelial injury and abnormal repair are central pathological events (25). Our findings extend this concept to HDM-related epithelial injury and suggest a potential association between dysregulated autophagic processing and allergen-related airway epithelial damage. Nevertheless, static measurements of LC3-II/LC3-I and p62 cannot directly establish impaired autophagic flux.

The mitochondrial abnormalities observed by TEM provide additional evidence of intracellular organelle injury. Mitochondrial swelling and cristae disruption are typical morphological signs of mitochondrial injury. When damaged mitochondria are not efficiently cleared, mitochondrial quality-control pathways may become overwhelmed, creating a cellular context that favors activation of the mitochondrial apoptotic pathway (26, 27). Yamada et al. recently highlighted the importance of mitochondrial quality control and mitophagy-related processes in shaping airway epithelial phenotypes and asthma outcomes (28). Although our study did not directly assess mitophagy, the coexistence of mitochondrial structural damage, autophagosome accumulation, and increased p62 levels suggests that HDM exposure may disturb epithelial mitochondrial homeostasis.

In addition to mitochondrial structural damage, JC-1 staining showed that HDM exposure induced mitochondrial depolarization. This change was accompanied by increased Bax and cleaved caspase-3 expression and reduced Bcl-2 expression, indicating a shift toward an apoptotic state in bronchial epithelial cells. These apoptotic changes are relevant to asthma pathobiology, as multiple forms of programmed cell death, including apoptosis, pyroptosis, ferroptosis, and necroptosis, have been implicated in disease progression (29). The concurrent alterations in autophagy-related markers, mitochondrial membrane potential, and apoptotic proteins suggest a potential link between dysregulated autophagic processing and epithelial apoptosis. This interpretation is supported by studies in other lung injury models. In silica nanoparticle-induced pulmonary fibrosis, Zhao et al. showed that blockade of autophagic flux in alveolar epithelial cells promoted apoptosis and aggravated fibrotic injury, whereas enhancement of autophagic degradation attenuated epithelial apoptosis (30). Evidence from tobacco-related lung injury models also supports a link between autophagy dysregulation and epithelial apoptosis, suggesting that impaired autophagic homeostasis can sensitize airway epithelial cells to environmental stress-induced injury (31, 32).

Several limitations should be acknowledged. Changes in the LC3-II/LC3-I ratio and p62 levels suggest autophagy dysregulation, but autophagic flux was not directly assessed. Future studies using lysosomal inhibitors and mRFP-GFP-LC3 reporter assays are needed to evaluate HDM-induced changes in autophagic flux. In the absence of functional intervention experiments (e.g., autophagy agonists/inhibitors, knockdown/overexpression of key autophagy genes), the causal relationship between autophagy dysregulation and epithelial apoptosis remains to be established. In addition, autophagy- and apoptosis-related markers were examined only in BEAS-2B cells and were not assessed in lung tissues. Therefore, whether the molecular alterations observed *in vitro* are reproduced in the airway epithelium of HDM-exposed mice or are associated with epithelial injury in the animal model remains to be determined. Finally, four mice were included in each group, which may have limited the statistical power of the animal experiments. Nevertheless, the marked and consistent histological differences support the preliminary conclusion that HDM exposure induces airway inflammation and mucus hypersecretion. Studies with larger animal cohorts are needed to confirm these findings.

In conclusion, our findings suggest that HDM-induced bronchial epithelial injury is associated with dysregulated autophagic processing and activation of apoptotic signaling. Although the causal relationship between autophagy dysregulation and apoptosis remains to be established, our findings suggest a potential link between altered autophagic homeostasis and epithelial injury following HDM exposure. Future studies are needed to determine whether restoring autophagic homeostasis can protect the airway epithelium during allergic airway inflammation.

## Data Availability

The original contributions presented in the study are included in the article/Supplementary Material, further inquiries can be directed to the corresponding authors.
